# Light pollution: a landscape-scale issue requiring cross-realm consideration

**DOI:** 10.14324/111.444/ucloe.000036

**Published:** 2022-06-29

**Authors:** Mariana Mayer-Pinto, Theresa M. Jones, Stephen E. Swearer, Kylie A. Robert, Damon Bolton, Anne E. Aulsebrook, Katherine A. Dafforn, Ashton L. Dickerson, Alicia M. Dimovski, Niki Hubbard, Lucy K. McLay, Kellie Pendoley, Alistair G.B. Poore, Michele Thums, Nikolas J. Willmott, Kaori Yokochi, Emily K. Fobert

**Affiliations:** 1 Centre for Marine Science and Innovation, Evolution and Ecology Research Centre, School of Biological, Earth and Environmental Science, University of New South Wales, Sydney, NSW 2052, Australia; 2 School of BioSciences, University of Melbourne, Parkville, VIC 3010, Australia; 3 National Centre for Coasts and Climate (NCCC), School of BioSciences, University of Melbourne, Parkville, VIC 3010, Australia; 4 Research Centre for Future Landscapes, School of Agriculture, Biomedicine and Environment, La Trobe University, VIC 3086, Australia; 5 Department of Behavioural Ecology and Evolutionary Genetics, Max Planck Institute for Ornithology, Seewiesen 82319, Germany; 6 School of Natural Sciences, Macquarie University, North Ryde, NSW 2109, Australia; 7 Agriculture Victoria Research, Bundoora, VIC 3083, Australia; 8 Pendoley Environmental Pty Ltd, 12A Pitt Way, Booragoon, WA 6154, Australia; 9 Australian Institute of Marine Science, Indian Ocean Marine Research Centre, University of Western Australia, Crawley, WA 6009, Australia; 10 Centre for Integrative Ecology, School of Life and Environmental Sciences, Deakin University, Burwood, VIC 3125, Australia; 11 College of Science and Engineering, Flinders University, Bedford Park, SA 5042, Australia

**Keywords:** ALAN, artificial light at night, light pollution, multi-disciplinary, adaptive management, ecological connectivity

## Abstract

Terrestrial, marine and freshwater realms are inherently linked through ecological, biogeochemical and/or physical processes. An understanding of these connections is critical to optimise management strategies and ensure the ongoing resilience of ecosystems. Artificial light at night (ALAN) is a global stressor that can profoundly affect a wide range of organisms and habitats and impact multiple realms. Despite this, current management practices for light pollution rarely consider connectivity between realms. Here we discuss the ways in which ALAN can have cross-realm impacts and provide case studies for each example discussed. We identified three main ways in which ALAN can affect two or more realms: 1) impacts on species that have life cycles and/or stages in two or more realms, such as diadromous fish that cross realms during ontogenetic migrations and many terrestrial insects that have juvenile phases of the life cycle in aquatic realms; 2) impacts on species interactions that occur across realm boundaries, and 3) impacts on transition zones or ecosystems such as mangroves and estuaries. We then propose a framework for cross-realm management of light pollution and discuss current challenges and potential solutions to increase the uptake of a cross-realm approach for ALAN management. We argue that the strengthening and formalisation of professional networks that involve academics, lighting practitioners, environmental managers and regulators that work in multiple realms is essential to provide an integrated approach to light pollution. Networks that have a strong multi-realm and multi-disciplinary focus are important as they enable a holistic understanding of issues related to ALAN.

## Introduction

Artificial light at night (ALAN) is a widespread anthropogenic pollutant that is rapidly increasing in intensity and global distribution. Current estimates suggest more than 80% of the human population, and nearly a quarter of the global land area, are exposed to light-polluted skies [1]. Consequently, ALAN affects most ecosystems globally, with the potential for profound impacts. At its core, ALAN alters natural light–dark cycles, disrupting a key driver of biological, ecological and evolutionary processes [2,3]. Emergent research has linked the presence of ALAN to altered physiology of plants [4] and animals [5]; shifts in activity patterns, behaviours, reproduction and survival of animals [6,7]; disruption of trophic and non-trophic species interactions [8,9]; and, significant changes to the structure of ecological communities [10,11]. The importance and severity of potential effects of this stressor are recognised across multiple taxa, habitats and ecosystems [7] and there is an increased desire to devise management strategies to minimise ecological impacts of ALAN.

A major challenge with mitigating the impacts of ALAN is that, while it is a global environmental pollutant [1] that damages ecological systems [7], it is also central to the functioning of modern human society [12]. However, beyond natural systems, ALAN can pose public health risks [13] and is energetically and economically costly [14]. Strategies to address the ecological challenges posed by ALAN therefore need to be interdisciplinary, involving researchers (e.g., ecologists, physiologists, social scientists, physicists), managers or regulators (e.g., local councils and government agencies) and practitioners (e.g., urban planners, developers, health specialists and lighting professionals). While interdisciplinary frameworks have been developed to foster collaboration among researchers, managers and practitioners to better manage urban lighting [15], they are largely applied within an individual realm, for example, marine, freshwater or terrestrial. We use the term ‘realm’ as defined by Bugnot et al. [16], to encompass a group of ecosystems that share common physical and ecological attributes (e.g., the marine realm includes all ecosystems present below the high tide mark while the terrestrial realm includes both air and land). Although realms are often considered as separate entities, they are intrinsically linked through ecological, biogeochemical and/or physical processes. Where these linkages are compromised, ecosystem functioning and services are affected and ecological systems may become less biodiverse and/or resilient to change [17,18]. Nevertheless, current management practices for light pollution do not consider connectivity between realms. The lack of a multiple-realm integrated approach means outcomes of practices are likely limited, at best, to small-scale, localised and/or temporary benefits [19].

In this paper, we review examples where ALAN affects two or more realms, directly and/or indirectly, and provide case studies for each example discussed. We identify three main ways in which ALAN can have cross-realm effects: through impacts on 1) species that have life cycles and/or stages in two or more realms, such as diadromous fish that cross realms during ontogenetic migrations and many terrestrial insects that have juvenile phases of the life cycle in aquatic realms; 2) species interactions that occur across realm boundaries; 3) transition zones or ecosystems such as mangroves and estuaries. We discuss the consequences of taking a single-realm approach to light pollution management and present a framework to help bridge this gap, incorporating both theoretical and empirical considerations. We also discuss existing challenges and hurdles to studying and managing light pollution. Given ALAN is projected to increase in all three realms in response to continuing human population growth [20], cross-realm management will be critical for ensuring the ongoing resilience of ecosystems [19].

## Potential paths for cross-realm impacts of ALAN

Mitigating the impacts of ALAN and prioritising appropriate conservation actions requires consideration of the fundamental interactions among realms (e.g., terrestrial, marine and freshwater) [17]. Shifts in ecological connectivity through the disruption of daily, seasonal or other cyclic movement of organisms or resources can have multi-realm consequences. For example, variation at the level of individual or population can affect food-webs directly but also influence functions such as pollination and nutrient cycling. These shifts, can in turn, have cross-realm implications due to trophic cascades and linked changes in ecosystem functions. This is particularly true if the organisms involved typically function across realm boundaries. Similarly, individual-level shifts can have cross-realm ecological consequences if the species in question has life histories or migratory patterns that traverse multiple realms, such as the two case studies we discuss below, salmon (freshwater juveniles, marine adults) and secondarily aquatic insects (aquatic juveniles, terrestrial adults). Throughout the paper, and in each case study presented, we outline known, measured impacts of ALAN, incorporate additional existing knowledge of species and/or habitats, and discuss how these may influence multiple realms.

Demonstrated impacts of ALAN include changes in the phenology, growth form and resource allocation of plants [4], as well as the behaviour, physiology, distribution and survival of animals [21–26]. There are multiple mechanisms underpinning these observed changes which may directly or indirectly affect other realms. For example, changes in the flux of inorganic and organic material (such as oxygen and nutrient fluxes), can directly impact land, sea and freshwater habitats [11,27], while indirect effects can be driven by bottom-up or top-down processes. For example, decreased diversity and abundance of aquatic insects due to ALAN is expected to affect terrestrial consumers that rely on aquatic prey, such as spiders, birds and bats [28,29]. Alternatively, changes may be driven by top-down processes, arising from, for example, shifts in the survival or behaviour of herbivores and/or predators. The consequences of such changes are varied and magnitude-dependent, but they can result in loss of biodiversity [30].

Transitional zones, such as estuaries and coastal wetlands, including the organisms that inhabit them, tend to be disproportionally affected by ALAN, because urban settlements, where ALAN is prevalent, are often developed near waterways [31]. Moreover, as these ecosystems are at the intersection of freshwater, marine and terrestrial realms, any ALAN effects are likely to have cross-realms consequences.

Rapid changes in the environment, including those linked to ALAN, can alter the environmental cues many animals use to select optimal habitats, resulting in them selecting sites that reduce their fitness [32,33]. These ‘ecological traps’ can promote disruptions or alterations in the movement patterns of organisms, resulting in increased risk of mortality and/or shifts in trophic interactions [34]. Ecological traps may not inherently have cross-realm impacts, however, as ALAN can disrupt species interactions or individual movements to create an ecological trap across more than one realm (see Box 1).

**Box 1. Light as an ecological trap**

Ecological traps arise when animals are attracted to and remain in poor-quality habitats where their fitness is compromised [32]. ALAN can cause ecological traps by influencing both the habitat selection decisions of animals and their fitness consequences. The orb-web spiders and aquatic insect community case study presented here clearly illustrates this – the adult stages of aquatic insects are attracted to artificial light where they suffer higher mortality because of the high density of webs. This case study provides further evidence of how ecological traps caused by ALAN can impact on cross-realm linkages. In this case, ALAN strengthens the magnitude of cross-realm predator–prey interactions. Specifically, the higher attraction and mortality of aquatic insects leads to increased aquatic-to-terrestrial subsidy flux [35].

Artificial light can also interfere with the migratory behaviour of species that occupy different realms as part of their life cycle. A well-known example of this is the impact of ALAN on the dispersal behaviour of sea-turtle hatchlings. Nocturnally emerging hatchlings are attracted to artificial lighting from coastal developments. Crawling towards an artificial light source can result in predation [36], impair their ability to swim offshore [37], leading to reduced rates of offshore migration and rates of transition between life stages [38].

Lastly, ALAN could increase cross-realm rates of disease transmission due to its impact on vector biology, such as biting mosquitoes. For example, in a recent study by Fyie et al. [39], artificial light masked natural daylength change which is the trigger for diapause, meaning mosquitos remained reproductively active for longer and produced more aquatic larvae. ALAN exposed mosquitos also had increased rates of blood feeding compared to control mosquitos. Given the preference for humans to associate with artificially lit environments at night, this suggests both changes in human and vector behaviour have resulted in a largely unrecognised ecological trap for humans.

Given the above, we have identified three broad pathways where ALAN can have cross-realm impacts: 1) for species that move across realms, through life cycles and/or stages or migratory patterns that occur in two or more realms, such as diadromous fish and many insects, as well as marine reptiles (e.g., turtles), mammals (e.g., seals) and birds (e.g., penguins and albatross) that are tied to land for breeding and/or resting; 2) where species interactions, such as predator–prey interactions, occur across realm boundaries; and 3) at transition zones or ecosystems such as coastal wetlands and estuaries, where multiple realms are inherently linked. These cross-realm linkages can be further affected if ALAN acts as an ecological trap (see Box 1). Below, we provide case studies of each way in which ALAN-related impacts can have cross-realm consequences.

### Impacts on species with life cycles/stages across two or more realms

The life cycles of many organisms occur in two or more realms. Examples include animals whose juveniles are aquatic while adults are predominantly marine or terrestrial, or marine animals that breed on land or in freshwater systems. Impacts of ALAN on any one stage are, therefore, predicted to have carry-over effects on subsequent life stages, consequently impacting different realms. We use two case studies to illustrate this, one on salmon (Salmonidae) and the other gives a broader overview of secondarily aquatic insects, such as dragonflies and mayflies.

#### Case study 1 – Salmon, a vector of energy and nutrients across realms

##### Demonstrated ALAN impacts

**Figure 1:**
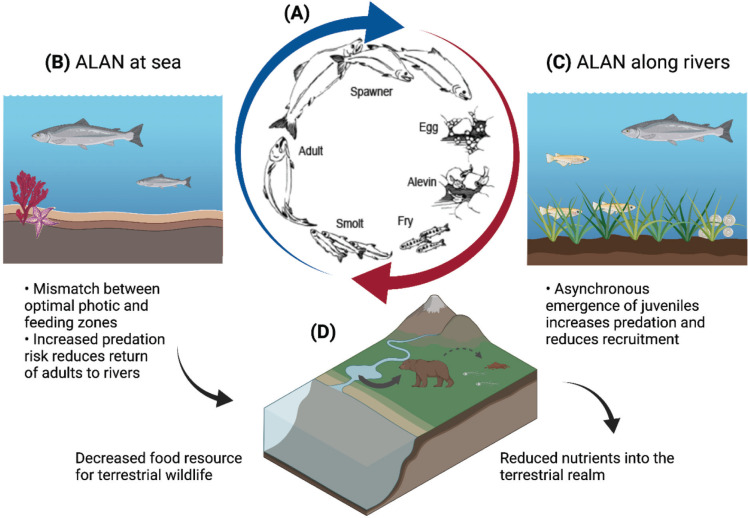
Schematic figure showing the potential cross-realm impacts of ALAN due to effects on different life stages in salmon. (A) Salmon spend their juvenile phase in rivers before migrating to sea to grow and mature. To complete their life cycle, they must return to the river to spawn. (B) ALAN at sea alters vertical movement of fish resulting in a mismatch between preferred light levels and optimal feeding zones. Additionally, ALAN results in increased predation of fish at sea and hence a decrease in adults returning to rivers. (C) ALAN along rivers disrupts synchronous emergence of juveniles resulting in increased predation which then reduces the recruitment of smolts out to sea. This reduction in adults returning to rivers and smolts migrating to sea results in trophic effects in both realms. (D) Illustrates one trophic effect in the terrestrial environment with reduced food resources for bears resulting in reduced nutrients into the terrestrial environment. Image created with BioRender.com.

Salmon, including the Atlantic (*Salmo salar*) and Pacific salmon (*Oncorhynchus* spp.), are anadromous fish – they spend their juvenile phase (e.g., alevins, fry and parr) in rivers, before migrating to the ocean as smolts (1–3-year-old juveniles that are physiologically adapted for sea water) to feed, grow and mature. Adults then return to freshwater systems for spawning (Fig. 1). ALAN has demonstrable impacts on several life stages of salmon species including fry [40,41] and smolts [42]. For example, the emergence of juvenile Atlantic salmon in streams is usually mediated by environmental cues, such as the presence of predators [43,44]. Fry are highly vulnerable to predation, and synchronous emergence can increase their chance of survival [45]. However, in freshwater river systems, ALAN is linked to asynchronous nocturnal emergence, disrupted dispersal and decreased weight of fry [40]. Experimental field evidence also demonstrated that smolt populations exposed to ALAN from streetlights along their native streams altered their migratory behaviour towards the sea, with potential consequences for their fitness and/or predation risk [42]. In the marine realm, ALAN associated with aquaculture practices, alters the vertical movement of smolt, resulting in potential trade-offs between preferred light and temperature levels, feeding and risk perception [46]. For example, surface mounted lights used in commercial farming induced movement of the smolt towards the surface, resulting in higher schooling densities and shallower nocturnal swimming depths compared to the day. This results in suboptimal environmental conditions and crowding of fish [47], with likely consequences to their growth and survival rates.

##### Potential cross-realm effects and knowledge gaps

Salmon are important vectors in transporting energy and nutrients between the ocean, freshwater and terrestrial environments [48] and thus the species-specific detrimental effects of ALAN may lead to broader cross-realm consequences. (i) Migrating adult salmon serve as a food resource for terrestrial wildlife as they travel upstream to spawn. Bears alone move up to 90% of all salmon biomass to land, sometimes hundreds of meters from their stream of origin [49]. Salmon-derived minerals and nutrients are further spread in the terrestrial environment through bear urine and faeces as these mammals move throughout the riparian and upland forests [50]. Salmon also support freshwater systems by providing nutrients from their carcasses following spawning [51] and play an important role in the marine food web during their migratory stage to the sea [48]. (ii) ALAN-associated impacts also have negative consequences for the total biomass of fish surviving to the ocean life stage: ALAN promotes asynchrony in the emergence of fry, likely increasing their predation risk [40] and reducing their survival [45]. Moreover, given the effects of artificial light on smolt dispersal, adult survival is also affected [42]. How much of salmon biomass is currently affected by ALAN and the magnitude of such effects for other realms remains unknown. Nevertheless, a study comparing 50 watersheds in British Columbia’s central coast in Canada showed that salmon influence nutrient loading to plants, shifting plant communities toward nutrient-rich species and declines in salmon will have the largest ecological effects on smaller and less productive streams [52]. Salmon populations are declining in many parts of the world due to a wide range of anthropogenic activities [44,53]. Management actions, however, rarely consider light pollution as a mitigating factor, and even fewer address cross-realm impacts. This is of concern given its cross-realm life history; efforts to mitigate the impacts of ALAN on salmon that are solely focused in one realm may be ineffective and economically wasteful if impacts from/in other realms are not considered.

#### Case study 2 – Aquatic insects (with terrestrial adults)

##### Demonstrated ALAN impacts

Dragonflies, mayflies and mosquitoes are classic examples of secondarily aquatic insects – those with an aquatic egg and juvenile phase and a terrestrial adult phase. The transition from the (often protracted) juvenile aquatic environment to the terrestrial adult environment is varied and taxon specific. For example, prior to their final moult, dragonfly nymphs typically move out of the water (usually at night) onto a branch or other structure where they eclose and emerge as air-breathing terrestrial adults. Mosquitoes remain in the aquatic environment emerging directly into the terrestrial environment as adults, typically remaining at the surface to allow their wings to dry and harden. Mayflies are hemimetabolous and thus do not have a pupal stage; instead, they emerge into the terrestrial environment as a winged subadult (or sub-imago) and then rapidly moult to adults.

The effect of variation in moonlight on adult insect activity has long been documented [49] and it is well recognised that artificial lighting is attractive to many adult insects – the behaviour is commonly exploited when trapping potential pests [55]. Recent evidence suggests sources of ALAN (such as streetlights) close to streams or water bodies may similarly change insect dispersal patterns (geographical or temporal) [35] and/or act as ecological traps for newly eclosing adults [56,57]. ALAN sources can also draw individuals away from the aquatic environment, an essential resource required for mating and egg laying [56,57], into suboptimal environments where the risk of mortality is increased [58]. Some species (e.g., dragonflies, mayflies and caddisflies) are also positively polarotactic, using horizontally polarised light to locate suitable water bodies for mating and egg laying [59]. In areas with anthropogenic sources of polarised light (reflected off asphalt surfaces, vertical glass and even vehicles), adult polarotactic behaviour can result in adults aggregating and females ovipositing on suboptimal non-aquatic surfaces, leading to reduced or no juvenile survival [60]. Moreover, anthropogenic sources of polarised light at night can also attract predatory insectivores, such as birds, lizards or spiders, resulting in increased adult insect mortality [61,62].

Even when eggs are laid in an appropriate body of water, the protracted aquatic juvenile phase may be vulnerable in the presence of ALAN. Evidence from other insects suggests aquatic juveniles may be directly attracted to external light sources, leading to shifts in foraging and other activity patterns [59] and possible increases in predation risk [64]. Moreover, experimental evidence from terrestrial invertebrates suggests prolonged exposure to ALAN during the protracted juvenile phase may influence growth, development and survival as adults [65–67].

##### Potential cross-realm effects and knowledge gaps

Secondarily aquatic insects are proposed as ideal bioindicators to assess the impact of cross-realm (aquatic and terrestrial) environmental change due to their sensitivity to anthropogenic stressors [68]. However, we lack direct evidence to confirm how impacts from one realm may influence the other. Moreover, there is surprisingly little information regarding the specific impact of ALAN on the independent life history stages of secondarily aquatic insects: in the largest review of urban impacts on dragonflies, ALAN was not even included [68]. For instance, the presence of anthropogenic sources of light are known to reduce reproductive success and increase predation rates of many secondarily aquatic insects (as stated above), but the degree to which exposure to ALAN results in selection of particular juvenile phenotypes that survive to the adult stage is unknown [3]. Ultimately, although many knowledge gaps exist, such insects form a large proportion of biomass and if ALAN affects their growth, survival and distribution, this is likely to have highly problematic outcomes that span multiple realms.

### Impacts on species interactions that involve two or more realms

The loss of, or changes in, species within a system can affect an entire cross-realm network, through altered competition and/or food web interactions, with unpredictable consequences for communities, ecosystems [69] and other, connected, realms [16]. Below, we highlight two case studies where observed or inferred effects of ALAN for one species or group are expected to affect multiple realms through species interactions and knock-on effects.

#### Case study 3 – Fishing bats: terrestrial mammals specialised for feeding in aquatic ecosystems

##### Demonstrated ALAN impacts

Worldwide, there are 16 species of fishing or trawling bats (e.g., from the genus *Myotis*). This group has ecological and foraging specialisations that make them reliant on both terrestrial and aquatic realms [70]. Fishing bats roost diurnally in caves, aqueducts, bridges, tunnels and tree cavities in the vicinity of water sources [71,72] and forage exclusively nocturnally on aquatic prey using their feet to trawl the surface of water for fish and aquatic insects [73–75]. Neither group of bats can detect submerged prey [76] and instead rely on echolocation of water surface irregularities created by fish and aquatic invertebrates [77].

**Figure 2:**
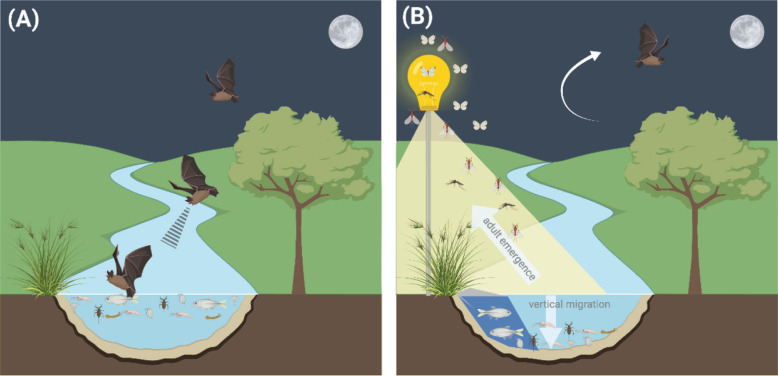
(A) Schematic figure depicting the aquatic ecosystem with fishing bats under natural light (B) and how artificial light at night influences prey species. As artificial light is introduced, aquatic prey species migrate into the shadows, sediment or to greater depths, making them unavailable to bats. Additionally, some aquatic insects emerge as aerial adult forms that are attracted to light. Fishing bats avoid lit areas and cannot switch foraging strategies to take advantage of the new aerial prey that is attracted to lights. Image created with BioRender.com.

ALAN has direct and indirect effects on the bat communities (Fig. 2). Of primary concern is the fact that fishing bats are largely light averse and thus either actively avoid lit areas, possibly due to increased risk of predation [78], and/or reduce their feeding attempts when waterways are lit [79]. Indirectly, light affects prey abundance. Aerial invertebrates are attracted to sources of ALAN, but fishing and trawling bats are unable to capitalise on this increased abundance due to their own aversion to light. Coupled with this, many aquatic invertebrates (potential prey items for bats) exhibit diel vertical migration: moving downwards from the water’s surface to deeper water during the day and moving upwards to the surface during the night [57,80] where they forage or potentially emerge as adult aerial invertebrates from the aquatic realm [35]. In areas exposed to ALAN, nocturnal vertical migration of invertebrates to the surface is reduced and fewer adults eclose, resulting in fewer opportunities for fishing bats to forage for prey.

##### Potential cross-realm effects and knowledge gaps

Light sources near aquatic habitats can therefore impact bats through impacts on their ability to forage. Again, the full consequences of this impact to fishing bat populations is unknown, and even smaller is our understanding on how population declines can in turn influence, more generally, their terrestrial habitats. However, due to the known association of these animals to both aquatic and terrestrial realms, knock-on effects on both are expected. Conservation and management efforts should thus include ALAN as a potential threat for these highly specialised species.

#### Case study 4 – Shifting energy flows between realms via impacts on orb-web spiders and aquatic insect communities

##### Demonstrated ALAN impacts

In riparian zones, increased predation pressure on emerging aquatic insects around ALAN through the attraction to nocturnal lighting by both predators and prey can reduce the transfer of biomass from aquatic to terrestrial systems. Short-term (two-month) exposure to ALAN was linked to an increased abundance and associated body mass of riparian long-jawed orb weavers (family Tetragnathidae) [81]. These effects were more pronounced for females compared to males and were concordant with greater numbers of prey items captured in spider webs under ALAN compared to webs under natural night-time conditions. However, a comparable, but longer-term, study (one year) found that although spider density initially increased (as in the previous study), there was a long-term decrease in spider density, as well as a decrease in the emergence of aquatic insects [82]. ALAN therefore shifts biomass from dark areas into artificially illuminated areas and dramatically shifts the distribution, overall abundance, and diversity of insect communities, reducing their abundance as prey for predators [22,35,81].

##### Potential cross-realm effects and knowledge gaps

By altering both the abundance and predation success of terrestrial predators, as well as the distribution and abundance of aquatic prey, ALAN can drive shifts in predator–prey interactions across realm boundaries, altering flows of energy between aquatic and terrestrial systems, with important consequences for both realms. Resource exchange from terrestrial to aquatic realms is an intrinsic facet of riparian habitats [28]. Spiders are important predators in riparian zones and can obtain more than 50% of their nutrition from aquatic sources, especially insects [83]. Therefore, the effects of ALAN on the diversity, abundance and distribution of spiders (both free-living and web-building), and/or the community of aquatic insects in riparian zones can alter cross-realm fluxes, with important regional and global implications for both terrestrial and aquatic realms [35]. The consequences of these effects of ALAN depend on the timescale considered and may be sex specific.

### Impacts on transition zones

In areas where light pollution affects critical transition zones (e.g., at ecosystem boundaries, affecting two or more realms) it is likely there will be consequences for ecosystems and function and service. Furthermore, transition zones tend to be disproportionally affected by ALAN, as many urban settings, where ALAN is prevalent, are developed near waterways [31]. Estuaries and coastal wetlands are critical transition zones that link freshwater habitats with marine and terrestrial environments [84]. These zones perform important ecological functions such as nutrient cycling and regulation of water and nutrient fluxes between realms [84].

Natural light at the air–water interface is a key factor linking terrestrial and aquatic realms. The amount of light that reaches the water surface in freshwater or coastal systems depends on the surrounding terrestrial habitat: structurally complex terrestrial environments, such as forested riparian zones, reduce the amount and colour of light reaching the water surface [85]. Species also vary extensively in their sensitivities to multiple light properties [86,87], and transition zones support several specialised species that have adapted to these complex lighting environments. For example, in estuaries with turbid waters, high loads of suspended material and low ambient light levels, fish species, such as the flathead grey mullet (*Mugil cephalus*), have evolved morphological traits that support dim-light (i.e., scotopic) vision, such as high rod density in the retina [29]. Similarly, the freshwater three-spine stickleback (*Gasterosteus aculeatus*) has a highly specialised visual sensitivity important for mate selection in both clear versus tannin-stained lakes [88]. Transition zones, therefore, are significant sites for understanding and managing cross-realm impacts of ALAN, both due to the vulnerability of organisms inhabiting these zones, and the prevalence of light pollution near waterways.

Shifts in the flow of resources in riparian zones – the interface between land and rivers or streams – can have impacts across multiple realms (see Case study 4). In their recent comprehensive review, Zapata et al. outlined a multitude of ways ALAN can specifically affect estuaries and highlighted potential cross-realm implications [29]. For example, ALAN-induced delays in the leaf fall of deciduous trees [4] can in turn reduce the input of nutrients from leaf detritus into aquatic systems, causing potential shifts in the biogeochemistry of aquatic systems [29]. Furthermore, in their review, Falcón discussed ALAN effects on riparian ecosystems [44] and Sullivan et al. recently demonstrated the impacts of ALAN on riparian systems through shifts in the community structure of invertebrates, consequently altering the flows of energy between aquatic and terrestrial systems [89]. Given these direct examples and published reviews of the impacts of ALAN on transition zones and flow-on effects across realms, we have not provided case studies here to further illustrate this mechanism. Instead, we want to highlight the importance of prioritising transition zones for management actions to limit the impacts of light pollution across multiple realms.

## Challenges and practical solutions for research and management of ALAN

**Figure 3:**
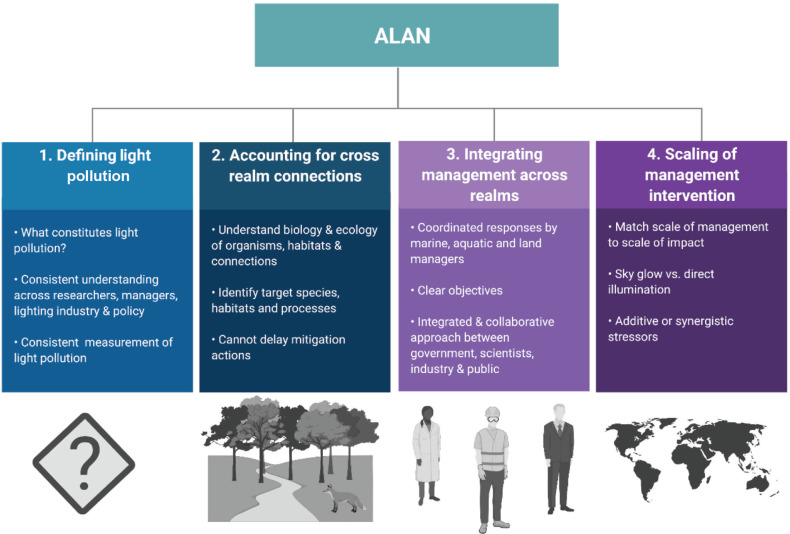
Proposed framework to explore the cross-realm impact of artificial light at night. 1. Defining light pollution – requires a shared understanding of what constitutes light pollution, and that its meaning and measurement is consistent across all stakeholders; 2. Accounting for cross-realm connections – requires knowledge of the ecological environment, the organisms, target species and how cross-realm impacts intersect; 3. Integrating effective cross-realm management – requires all stakeholders to be clear on objectives and outcomes; and 4. Effective scaling of management integrations – requires the scale of the management intervention to match the scale of impact. Image created with BioRender.com.

Several challenges exist that need to be addressed for the impacts of light pollution to be effectively understood and managed, both within and across realms. A major difficulty (and potential point of contention) encountered when dealing with cross-realm issues is determining the boundaries for management and governance [90]. For example, land-based sources of ALAN may indirectly influence the productivity of aquatic systems through its impact on nutrient inputs from terrestrial sources through, for example, changes in the leaf fall patterns of deciduous trees. In this case, areas are separated by physical and jurisdictional boundaries (e.g., land and coastal managers) and potentially social boundaries (different communities or social networks). Here, we propose a framework for cross-realm management, which builds on previous frameworks for conservation and management across realms [17,19,91,92], but with a specific focus on light pollution (Fig. 3).

### Challenges and practical solutions

#### Defining light pollution

One of the main challenges for driving practical solutions to manage ALAN is agreeing to a collective understanding of how and when lighting should be defined as pollution [93]. Here, we define light pollution as light introduced into the environment by humans at intensities that are higher than the natural level at that time for the given environment and that has the potential to cause harm to humans and/or the environment. In a recent analysis, Schulte-Römer found that light pollution experts (including scientists and managers) had a stronger and more consistent view of what constitutes light pollution than lighting professionals (such as lighting designers, urban planners and engineers) [93]. Importantly, however, both groups had very skewed views when considering potential issues caused by light in areas where it is ‘unwanted’, depending on the habitat or realm. Of the respondents identified as light pollution experts (n = 89), approximately 90% considered light to be pollution when it obscures the visibility of stars, or when fixtures were installed close to observatories. In contrast, only 66% of experts surveyed considered lighting as pollution when it was installed close to bodies of water. Among the respondents identified as lighting professionals (a total of n = 67 respondents), this dropped to only 17%. These results highlight a common misconception, and a massive global problem, namely, that light is a ‘land’ problem rather than of fundamental significance for all ecosystems on Earth. These findings also ignore the critical need for fluctuating light levels (both day and night) that have characterised the evolutionary history of that life. Therefore, the first steps to successfully managing light pollution within and across realms are to (i) raise awareness of the importance of fluctuating light regimes for ecological process; (ii) enhance understanding of the impacts of artificial light across all realms: terrestrial, freshwater and marine environments; (iii) broaden knowledge regarding the impact that light within one realm can have for biodiversity and ecosystem function within other realms and (iv) understand the ‘acceptable’ levels of ALAN for both the local ecological communities and society (i.e., trade-offs between ecological impacts and societal needs or desires). Critically, this needs to include multiple stakeholders, including the general public.

#### Accounting for cross-realm connections

The next step in managing light pollution across realms is to understand the biology and ecology of organisms and habitats of interest and their potential linkages, so that management interventions can account for connections across realms in a more comprehensive way. Ideally, the extent of the impact of ALAN on target individuals, populations, habitats and systems, as well as the mechanisms driving these changes, will be well-known within and across realms. However, we acknowledge that, unfortunately, the current state of habitat degradation worldwide and rapid expansion of ALAN means that we cannot afford delaying mitigation actions until the impacts, or even the potential unintended risks of management interventions, are completely understood [107]. Therefore, we need to keep gathering the – still much needed – scientific information on the effects of ALAN, within and across realms, while, at the same time, implementing local, regional and global best practice guidelines to prevent or reduce such impacts (see discussion in Box 2).

**Box 2. Cross-realm exploitation of resources using artificial light at night**

Artificial light at night is known to attract and/or aggregate many organisms. This effect can be exploited by predator species within and across realms, if, for example, a terrestrial predator is exploiting an aggregation of aquatic organisms to a light source. One of the best cross-realm examples of how ALAN can be used to exploit resources is the use of artificial light by humans during night-time fishing.

The attraction of many fish and aquatic invertebrates to light has been known for thousands of years, and artificial light has been used by humans to improve fishing efficacy for centuries [94]. Light at night attracts small fish, insects and/or plankton through positive phototaxis, disorientation or curiosity [95], which in turn attracts larger predatory fish and invertebrates [96]. Historically, humans exploited this behaviour by lighting a fire on a beach to attract fish into the shallows to facilitate harvesting (e.g., by spearing or netting) [94]. Today, incandescent, fluorescent, metal halide and LED above water and underwater lights are used for artisanal and industrialised fishing practices worldwide to increase harvesting [97,98]. In fact, certain fisheries cannot operate effectively without the use of lights, such as the squid jigging fishery. Jigging for squid dates back to antiquity in many parts of the world; however, in the recent century, the addition of artificial light to jigging gear has substantially increased landings due to the effect of light at night on attracting and concentrating squid [97].

The effects of ALAN on fish attraction/aggregation are not lost on recreational fishers; recreational fishers often target artificially lit areas for night fishing, as they know certain target game species will follow baitfish into the illuminated areas [99]. Urbanisation has led to an increase in artificial light installations in coastal areas, illuminating a substantial portion of shallow aquatic habitats at night [100,101], and has therefore created ample opportunities for recreational fishers to exploit artificial lighting (i.e., light pollution) to increase catch rates.

The increased harvest resulting from fishing practices using ALAN can lead to overfishing and increased rates of bycatch in a fishery which may have negative impacts on fished populations (e.g., reduction in size and altered life-history traits) [97] and thus ecological consequences for the marine or freshwater realms (e.g., through trophic cascades). However, as responses to ALAN are species-specific, ALAN can be used by humans to both increase fishing harvest and reduce catch rates of different species. The use of artificial light has been recognised as a potential tool for bycatch reduction in commercial fisheries, and therefore ALAN can also be exploited to mitigate cross-realm impacts through minimising effects of fishing on non-target organisms. Research on the use of artificial light to reduce bycatch has demonstrated varying levels of success (e.g., [102–104]) and is dependent on species of interest, light properties tested and proper placement/location of (often LED) lights within the fishing gear. However, the use of artificial light to deter adult sea turtles has also proved to be effective [105,106], resulting in LED lights now widely applied worldwide in pelagic gillnet fisheries to reduce sea turtle bycatch [98]. This positive use of artificial light demonstrates that with species-specific knowledge, it is possible to harness the effects of ALAN for positive impacts across realms.

#### Integrating management across realms

A key challenge associated with managing the impact of ALAN across realms is the lack of collaboration between different stakeholders and the existence of methodological disparities across realms. The compartmentalisation that can exist within governance structures, such as within and between local, state/territory and federal government agencies, inevitably generates a lack of consistency in management decisions which is exacerbated when considerations involve multiple realms (and thus multiple stakeholders). Contributing factors include poor communication, differing and potentially competing priorities and a lack of collaboration among the sectors and agencies responsible for planning and environmental protection in the different realms; a lack of spatial data on cross-realm processes; and, logistical difficulties associated with adapting existing decision-tools and coordinating different governance systems to fit the current purpose [90, and references therein].

To successfully implement cross-realm management strategies, some key general steps (adopted and modified from e.g., [16,19,108], can be taken. First and foremost, a clear objective regarding the desired outcomes is necessary. For issues pertaining to light pollution, these can include minimising or eliminating the effects of ALAN on ecologically, culturally and/or commercially important target species/groups or a target area (e.g., a transition zone, migratory pathways or a protected area). This necessitates an integrated and collaborative approach with policy makers, regulators, scientists, lighting designers, developers and the general community, including First Nations People, to identify potential conflicting interests and devise solutions accordingly. Ultimately, we need to both unify terminologies and agree on desired outcomes [16,109], and, ideally, understand potential thresholds of ‘acceptable’ artificial light levels across different species and realms, which will likely involve a compromise between levels of ecological impacts caused by ALAN and societal needs or desires.

Determining ALAN thresholds, however, requires standardised measurements of light. Currently, there is great inconsistency in instrumentation and light parameters within and across realms. Discrepancies in lighting measurements exist for valid and practical reasons – for example, the measurement and instrument used needs to match the scale of both the light pollution being measured (i.e., direct sources of light vs. skyglow) and the ecological or biological response of interest (e.g., insect attraction to a streetlight vs. bird migration). Moreover, as far as we know, there is not yet available affordable and easy-to-use instrumentation to adequately measure light levels under water. However, there is a clear and urgent need to standardise, where possible, the measurement of light pollution, so that outcomes are comparable and applicable across realms (see Box 3 for further discussion). It is important to note, however, that knowing relevant light ‘levels’ is not enough for effective management for ecological outcomes. At the extreme, any light that is not natural in its origin is likely to interfere with ecological processes. Thus, perhaps of greater importance, we need to be able to measure and understand how light properties (including spectra and intensity) affect organisms and habitats in multiple realms. Standardising how and which properties of light are measured will facilitate communication of clear and specific recommendations (including biologically relevant thresholds) between researchers, practitioners and managers. This will permit informed decision-making when considering potential impacts across different habitats and realms and allow better assessment of the risks when night-time illumination is unavoidable and/or socially desirable.

**Box 3. Discrepancies in light measurements**

A complicating factor influencing the ability of scientists to confidently predict the impact of light on a sensitive receptor is the lack of an agreed upon standard method for modelling, measuring and monitoring light or skyglow [110–112]. Instrument types and applications vary widely: instruments include lux meters, spectrometers and cameras which measure light emitted directly from a source or light reflected from a surface, from overhead looking down on the earth (satellite based) or from the ground looking up or horizontally across the landscape. Limitations include: restrictions in the wavelengths they measure (i.e., they do not measure all wavelengths across the entire visible spectrum), detection limits that are not low enough to measure sky glow or intensities that elicit a biological response, highly technical instruments requiring specialised knowledge to operate and maintain, and a wide range of different measurement units.

Arguably, many of the existent ‘disparities’ arise because different instruments are designed to measure different things, depending on the objectives of the users. For example, studies aiming to measure large-scale environmental effects due to sky glow will (and should) measure different variables (and consequently use different instruments) than studies where the primary aim is to evaluate the effects of streetlight on one species of insect. Nevertheless, whenever possible, studies with similar objectives and/or operating at similar spatial scales, should try to standardise measurements. Crucially it is important to understand the operating limits of even the simplest instruments, as instruments can be misused or used for an inappropriate environment [113]. Similarly, the literature acknowledges that there are no conclusive intensity thresholds below which artificial light is not harmful to species and habitats [114], and even the low intensity light characteristic of skyglow can affect organisms [115,116].

Attempts to compare or standardise measurements across realms adds further complications. For instance, while remote sensing techniques are commonly used as a best proxy to quantify the amount of artificial light at night on terrestrial systems, there are serious challenges associated with the use of this technology in water bodies/underwater (see the extensive discussion in [110]). Furthermore, different disciplines often use different physical quantities and units for measuring light, creating confusion even among experts [110]. For instance, much of the existing data on the quantity and quality of light reaching both terrestrial and aquatic systems assess different physical parameters (spectral irradiance, illuminance); have used several different instruments to acquire measurements (e.g., SQM, luxmeter, spectrometer, digital camera); and report outcomes using different measurement units (lux, candela, magnitudes, Watts). Therefore, as stated by [110], ‘there is no clear coherence between these measurements, although each of them was well designed and conducted’. Cross-realm assessment and management of light pollution is impeded by the discrepancies in measurements of light pollution across systems and disciplines. However, standardisation of measurements across species level responses, systems and realms of interest is incredibly challenging, as measurements currently generally differ for valid, practical reasons, such as the ecological and spatial scale of interest. This challenge highlights the value of cross-realm and cross-discipline networks for developing solutions that allow efficient conservation and management actions across species, habitats and realms.

#### Scaling of management intervention

Ultimately, there is a need to match the scale of the management intervention to the scale of impact [19]. Light pollution impacts occur at the landscape scale, and include impacts caused by sky glow, light scattered in the atmosphere [1,116] and those caused by direct illuminance from light sources (e.g., streetlights). Impacts caused by direct illuminance are, in theory, easier to mitigate than impacts caused by sky glow – which can be an issue even tens (and possibly hundreds) of kilometres from urban light sources [85] and require management interventions at much larger, landscape level, scales to prevent or mitigate cross-realm impacts. For example, research has shown that light pollution can spill into otherwise protected areas up to 15 km from urban centres [117]. Additionally, a recent study has highlighted the potential for synergistic interactions between sky glow and direct illuminance (Dickerson et al., unpublished data). Management actions therefore need to consider, whenever possible, multiple spatial scales to mitigate light pollution and avoid cross-realm impacts. Extensive examples on specific interventions and management strategies can be found in the literature [86,119].

Light pollution is just one of a multitude of anthropogenic stressors associated with urbanisation [120], which can also cross realm boundaries. Therefore, management interventions should also consider potential additive or interacting impacts from multiple stressors [121]. For example, ALAN and night-time warming have non-additive interactive effects on the predation of aphids by lady beetles, decreasing aphid population densities [122]. Similarly, particular traits in birds can be impacted by both ALAN and noise pollution: light pollution is associated with advancement in reproductive phenology of several species of birds while noise decreased clutch size of closed-habitat (i.e., forests) birds [123]. Interactive effects of anthropogenic stressors with ALAN, however, remain poorly understood [44]. Understanding, or at a minimum identifying, other stressors that may interact with or act simultaneously with ALAN will enhance cross-realm management outcomes. Moreover, climate change adds additional challenges to cross-realm studies as it increasingly modifies key land–sea ecological and social processes, therefore increasing the urgency for transboundary management initiatives.

### Cross-realm management success

Few example exist of successful of management of ALAN which have resulted in a reduction of cross-realm impacts, and most of these examples involved management interventions that targeted a single species rather than an assessment at community or ecosystem levels. Successful examples include: 1) the mitigation of impacts on shearwaters (Phillip Island, Victoria, Australia) through changes to the timing and colour of street lights, particularly during critical periods of the life cycle – that is, fledging [124,125]; and 2) legislation related to nesting marine turtles [119]. Below, we expand on the latter.

Marine turtles have complex life histories that cross marine and terrestrial realms and are considered key indicators of ecosystem health [126]. Light pollution can reduce the reproductive viability of turtle stocks by disrupting critical behaviour such as the ability of hatchling marine turtles to successfully reach the ocean [127]. Light in nearshore waters (e.g., boats at anchor, jetties or coastal lighting) can influence the offshore dispersal of hatchlings in the critical minutes and hours after they leave the beach. Attraction to artificial lights increases the time hatchlings spend crossing predator-rich nearshore waters before reaching the safety of deep water offshore, thus increasing their vulnerability to predation [128–130]; and as predators are also attracted to the same lights, predation pressure can be high. In Australia, activities that involve artificial light at night that is likely to impact marine turtles must be referred for environmental assessment. Proponents must demonstrate, via formal risk assessments, how the impact of ALAN on all age classes of marine turtles will be mitigated and adaptively managed. Mitigation measures that benefit marine turtles have been summarised in the National Light Pollution Guidelines for Wildlife Including Marine Turtles, Seabirds and Migratory Shorebirds [119] and include 1) management of the physical aspects of the light, such as intensity (lumen output), colour (wavelength) and elevation above dark horizons behind the beach, 2) the maintenance of dark zones between turtle nesting beaches and light sources, and 3) shielding and targeting of light fixtures to avoid direct visibility and limiting sky glow [119]. Given light pollution sources that can affect turtles can be both marine and terrestrial, management actions in both realms are likely required, with the collaboration of terrestrial and aquatic ecologists and lighting professionals, to successfully avoid terrestrial–aquatic impacts.

It is important to note, however, that, even though the management actions outlined here were focused on one particular group of organisms (e.g., marine turtles), a general understanding of both the terrestrial and marine realms and potential linkages among them, as well as a clear desired outcome, were necessary to devise efficient strategies. None of which could have been achieved without collaboration among different stakeholders in each individual realm.

## Mitigating impacts of future lighting

There is increasing recognition that conservation and management strategies should be designed to account for cross-realm connections [19,131]. A recent study developed a national-scale conservation framework that incorporated linkages among the marine, freshwater and terrestrial realms, to select protected areas for minimising the threats of both land-use and climate change [131]. The cross-realm approach resulted in changes to both terrestrial and marine priorities compared to when connections among realms were not considered. The authors also argued that a cross-realm approach allowed the identification of potential trade-offs and opportunity costs of conservation versus ecological benefits, as well as the implementation of interventions with multiple objectives (such as habitat management and biodiversity protection) [131].

Increasing the uptake of a cross-realm management approach requires increased and improved communication between researchers, lighting practitioners, managers and regulators that work within and across different realms. The creation of professional networks is a great way to begin such conversations. In Australia, the Network for Ecological Research on Artificial Light (NERAL; www.neralaus.com) was established to provide a platform to connect researchers and practitioners working towards mitigating the impacts of light pollution within and across realms. NERAL is a professional network of academic scientists and consultants, with a wide range of expertise, including terrestrial and marine ecologists and physiologists, and managers from local and federal government agencies. A primary aim of the network is to increase communication between scientists and managers working on different species, habitats and/or realms. This will allow: 1) managers to easily access information crucial to developing and implementing interventions to prevent or mitigate light pollution impacts, and 2) researchers to identify management priorities and provide evidence-based information to shape management interventions. Networks that have a strong multi-realm focus such as NERAL are important, as they enable a more holistic understanding of issues related to ALAN. They can also provide an opportunity to develop standardised methods for measuring light so that the impacts can be compared across realms. This holistic approach can then be translated into the ongoing implementation of strategies to reduce impacts of ALAN across terrestrial, marine and freshwater realms.
